# An Online HIV Self-Sampling Strategy for Gay, Bisexual and Other Men Who Have Sex with Men and Trans Women in Spain

**DOI:** 10.1007/s10900-023-01311-8

**Published:** 2023-12-23

**Authors:** Héctor Martínez-Riveros, Yesika Díaz, Marcos Montoro-Fernandez, Sergio Moreno-Fornés, Victoria González, Esteve Muntada, Pol Romano-deGea, Rafael Muñoz, Juan Hoyos, Jordi Casabona, Cristina Agustí

**Affiliations:** 1 Doctorate Program in Methodology of Biomedical Research and Public Health, Department of Paediatrics, Obstetrics and Gynaecology and Preventive Medicine, Universitat Autònoma de Barcelona, Badalona, Spain; 2 Centre for Epidemiological Studies on Sexually Transmitted Infections and AIDS of Catalonia (CEEISCAT), Ministry of Health of the Government of Catalonia, Badalona, Spain; 3 Germans Trias i Pujol Research Institute (IGTP), Campus Can Ruti, Badalona, Spain; 4 Fundació Institut d’Investigació en Ciències de la Salut Germans Trias i Pujol (IGTP), Edifici Muntanya, Carretera de Can Ruti, Camí de les Escoles s/n, 08916 Badalona, Spain; 5 Biomedical Research Center Network for Epidemiology and Public Health (CIBERESP), Instituto de Salud Carlos III, Madrid, Spain; 6 Microbiology Service, Clinical Laboratory Metropolitana Nord, Badalona, Barcelona, Spain; 7 Early Detection of Cancer Research Group, EPIBELL Program, Bellvitge Biomedical Research Institute, L’Hospitalet de Llobregat, Barcelona, Spain; 8 Independent consultant, Madrid, Spain; 9 Department of Genetics and Microbiology, Autonomous University of Barcelona, Badalona, Spain

**Keywords:** HIV, GBMSM, Public health, Transgender, Self-sampling, Home-based

## Abstract

We aimed to evaluate the feasibility of an online self-sampling pilot intervention for HIV testing addressed to gay, bisexual, and other men who have sex with men (GBMSM) and trans women (TW) users of dating apps in Spain. The website https://www.testate.org/ was designed to offer self-sampling kits for HIV testing and online consultation of the results. It was advertised on gay dating apps. Participants requested the delivery of a saliva self-sampling kit by mail and a postage-paid envelope to send the sample to the reference laboratory. An anonymous acceptability survey was conducted. The cascade of care was estimated. From November 2018 to December 2021, 4623 individual users ordered self-sampling kits, 3097 returned an oral fluid sample to the reference laboratory (67.5% return rate). 87 reactive results were detected. 76 were confirmed to be HIV-positive, we estimated an HIV prevalence of 2.45% (95% CI 1.9–3.0%). 100% of those referred to specialized care are in treatment. 44.3% (44.27%) of participants took more than one test. 23 incident cases were detected among repeat testers, of which 20 were confirmed. The estimated incidence was 1.00 confirmed case per 100 individual-years of follow-up. 98.01% of participants would recommend it to a friend. The most identified advantages were convenience and privacy. We demonstrated that the online offer of oral self-sampling kits for HIV detection and reporting results online among GBMSM and TW users of dating apps is feasible. The intervention counted with a high acceptability and high efficacy (in terms of reactivity, confirmation and linkage to care rates).

**Supplementary Information:**

The online version contains supplementary material available at 10.1007/s10900-023-01311-8.

## Introduction

Globally, gay, bisexual and other men who have sex with men (GBMSM) accounted for 21% of new HIV cases in 2021. If we discount cases detected in sub-Saharan Africa, this percentage rises to 41%. According to UNAIDS, GBMSM have a 28 times higher relative risk of acquiring HIV infection than adult men in the general population [1].

Transgender people, especially transgender women (TW), are at higher risk of HIV infection [2]. According to a meta-analysis, TW have a pooled prevalence of HIV infection of 19.1% (95% CI 17.4–20.7). In high-income countries it increases to 21.6% (95% CI 18.8–24.3) [3]. According to UNAIDS, TW are 14 times more at risk of acquiring HIV than adult women in the general population [1].

In 2021, 2786 new HIV cases were reported and 340 AIDS cases were diagnosed in Spain [4]. Men accounted for 86.1% of new HIV diagnoses and the median age at diagnosis was 36 years [4]. Although HIV testing in Spain is offered free of charge at all levels of the health system, 49.8% of new diagnoses reported in 2021 were late diagnoses (< 350 CD4 cells) [4]. Late diagnosis (LD) is associated with increased morbidity, mortality and higher economic costs, as well as a longer period of transmissibility and thus a greater contribution to HIV incidence [5–8]. GBMSM, at 43.4%, have the lowest LD; however, given their weight in the overall numbers, they are the largest group of late-diagnosed HIV cases (52.4% of the total) [4].

There are also particularities of the sexual culture among GBMSM, such as the use of new technologies to find sexual partners, the globalisation of risky sexual practices and recreational drug use during sex, which may influence the spread of infection [9]. The European Men Who Have Sex with Men Internet Survey (EMIS) showed that 60.4% of GBMSM in Spain did not use a condom during their last sexual intercourse with a non-stable partner, 14.1% used drugs before or during sex and 19.4% of GBMSM had never been tested for HIV [10]. Reducing the number of undiagnosed infections and early treatment of these individuals is a priority because it would also have an impact on HIV incidence [11, 12]. National and international guidelines recommend that GBMSM should be tested annually and every three months for those at risk, with a history of STIs or taking pre-exposure prophylaxis (PrEP) for HIV [13–15]. In order to increase access and frequency of testing in this key population, different screening strategies have been developed outside healthcare settings. Interventions in gay venues such as bars, clubs and saunas [16, 17], as well as community-based voluntary counselling and testing services (CBVCT) [18], have proven successful in enabling access to HIV testing in this population.

Complementary testing modalities to existing testing strategies, such as self-testing and self-sampling, are important options to diversify and optimise access to testing. They are recommended by international bodies such as the WHO and ECDC [14, 19] should be regulated and made available as part of national policy and practice.

Digital technologies are increasingly used to deliver sexual health interventions [20], including internet-based STI testing (electronic STI testing). It allows users to order a test kit via a website or app, collect their own samples, return them to a laboratory, and receive notification of results by text message, phone or email [21]. Transferring tasks to patients through virtual services has been shown to be cost-effective [22]. Previous studies have shown that electronic STI testing services increase the uptake of STI testing, including HIV, in all groups, including high-risk groups [23–25].

The popularisation of smartphone use and the emergence of location-based real-time dating apps (e.g. Scruff, Grindr and Romeo) have transformed traditional avenues of socialising and promoted new ways of meeting and engaging with potential romantic and/or sexual partners [26]. Location-based real-time dating apps are very popular among the GBMSM community [27]; in Spain, 73% of GBMSM met their last non-stable partner online [10]. Previous work has shown that GBMSM who use these apps tend to have more sexual encounters, more frequent anal intercourse, more unprotected sex and a higher number of sexual partners known to have HIV and other STIs [27–31]. In the city of Barcelona they have shown that the use of dating apps was significantly associated with younger, university-educated GBMSH, high number of sexual partners, lower condom use and practicing chemsex [32].

There is a need to explore new ways to access this population with more risk factors for HIV and other STIs. The TESTATE (*TESTATE is the Spanish word for describe: Test yourself*) platform [33] launched in November 2018 is a pilot online self-sampling intervention for HIV testing and online consultation of results aimed at GBMSM and transgender dating app users in Spain. The objectives of the study were to describe the socio-epidemiological characteristics of participants, assess its feasibility by describing its effectiveness, satisfaction and willingness, estimate the prevalence of HIV infection and identify possible factors associated with HIV infection.

## Methods

### Study Design and Inclusion Criteria

The pilot intervention consisted of offering HIV self-sampling test kits through a secure website and online consultation of test results. The study focused on two different key populations: GBMSM and TW who were users of real-time location-based dating apps.

This prospective, non-randomised study included GBMSM and TW ≥ 18 years old living in Spain and who agreed to sign the informed consent form.

### Recruitment

The intervention was disseminated through the following GBHSH and TW dating apps: Grindr, Scruff, Wapo, PlanetRomeo, Bakala, MachoBB, Trans4men, Telechapero. Through intermittent campaigns, participants were invited to participate through personal messages and promotional banners. The study period was between November 2018 and December 2021.

### Web-Based Intervention and Participatory Process

The web-based intervention was based on the TESTATE project (https://testate.org/). The procedure consists of five steps (Supplementary Figure).

Firstly, respondents accessed the study website (https://testate.org/), enrolled and accepted an online informed consent form. All participants were provided with online information about the study and were given the opportunity to ask questions and clarify doubts to the study coordinator by email or telephone.

Secondly, participants requested a free HIV self-sampling kit by providing contact details such as first name, surname, mobile phone number and main postal address. All identifying information collected on the website was encrypted. Participants then completed an online survey on socio-demographic data such as age, sex, gender, country of birth, size of city of residence, sexual orientation, HIV testing history, STIs diagnosed in the past 5 years, condom use, number of sexual partners in the past 12 months and PrEP use.

Thirdly, the self-sampling kits were sent in a white envelope with no return address information. Participants then had their samples collected. Included in the kits was an oral swab to collect saliva for HIV 1+2 antibodies (ORACOL Saliva Collection Device, Malvern Medical Developments, Worcester, UK) and a pre-paid envelope for shipment to the reference laboratory. The kits also included an illustrated leaflet with guidance on how to collect the sample. A video demonstration of sample collection was available on YouTube.

Fourthly, participants sent samples to the laboratory and test results were delivered online via the study website. Participants received a text message (SMS) informing them of the availability of their results and how to view them. SMS reminders were sent to participants who did not check their results. Participants with a negative result were invited to receive an SMS reminder to repeat the test at 3/6/12 months. Participants with a reactive result were followed up individually.

Finally, two weeks after consulting their result, an anonymous survey was sent by email to all participants, collecting the following data: evaluation of the experience from 1 to 5, would repeat the experience, would recommend it to a friend, perceived advantages and disadvantages, and preferred way of receiving the test results.

### Laboratory Methods

Oral fluid samples were tested with the Genscreen HIV1+2 enzyme immunoassay (EIA) technique (BioRad Laboratories, Inc., Hercules, United States of America) [34] for the detection of HIV1+2 antibodies in the Microbiology Service. Metropolitana Norte Clinical Laboratory. Hospital Universitari Germans Trias i Pujol, Badalona, Barcelona.

### Follow-Up of Reactive Participants

All participants with a reactive result were asked to visit their general practitioner (GP) or a CBVCT service for confirmation. After 6 weeks, these participants were contacted by telephone to ask if they had confirmed their result, the location, date and result of the confirmation; and if they had been referred to a specialised HIV unit.

### Evaluation of the Intervention

We assessed the feasibility of the TESTATE HIV screening strategy among its users based on a conceptual framework adapted from previous models [35, 36]. The adapted framework divides the concept of feasibility into the following subdomains: effectiveness, satisfaction and willingness. Efficacy was defined as the ability of participants to make the effort and take the time to order the self-sampling kit, obtain the sample, send it to the reference laboratory and consult the results online, as well as to follow the linkage procedure to health care if necessary. Satisfaction was described as the feeling that getting tested for HIV through the TESTATE intervention was convenient and that it is a process they would experience again. Readiness was defined as the participants’ intention to follow the entire procedure and the number of individuals who repeated the test.

For the evaluation of the whole intervention, and taking into account the objectives of the study, 14 cases that were HIV+ at the time of the kit request were excluded from the analysis.

The number of individuals with a reactive result among all individuals tested (reactivity rate) was calculated. Among them we calculated: the number of individuals with a false reactive result (defined as individuals with a reactive result and a negative result of the confirmatory test—Elisa test on a blood sample), individuals with an unconfirmed result (individuals with no information on the confirmation of their reactive result), and newly confirmed HIV diagnosis (individuals with a reactive result and a positive result of the confirmatory test—Elisa and Western Blot test on a blood sample). HIV prevalence was estimated by calculating the proportion of confirmed HIV-positive individuals out of the total number of individuals with at least one returned sample. A 95% confidence interval was calculated.

The linkage to care rate was assessed by calculating the proportion of people reporting a confirmed HIV diagnosis who had been linked to a specialised HIV unit.

The percentage of participants who repeated HIV testing throughout the intervention was calculated, as well as the median and interquartile range of test repeats per individual.

HIV incidence was estimated by considering those individuals who had more than one test result. Confirmed positive cases were considered and incidence was estimated per 100 person-years of follow-up. Follow-up time was defined as the time from the first sample request to the last result consultation date.

A descriptive analysis was conducted comparing socio-demographic characteristics, risk behaviours and previous STI diagnoses between reactive and negative participants. Categorical variables were compared using Pearson’s χ^2^ test. Comparisons of quantitative variables were made between 2 or more groups using non-parametric tests (Kruskal–Wallis). The multivariate logistic model and the negative binomial model were used to estimate factors associated with obtaining a reactive result and test repetition across the intervention, respectively. A significance level of 5% was considered for all analyses. All analyses were conducted using R version 4.0.5.

The HIV care cascade was calculated. As a first step we included the total number of participants who requested a self-sampling kit through the TESTATE intervention. Secondly, we calculated the proportion of individuals who returned at least one sample to the reference laboratory. Thirdly, we calculated the proportion of individuals with a reactive result who were not known to be HIV-positive and were not false positives. Fourthly, we calculated the proportion of individuals with a reactive result who confirmed their result. Finally, we calculated the proportion of individuals diagnosed with HIV who were linked to care at a specialised HIV unit. Each stage of the cascade was calculated using the previous stage as the denominator.

## Results

**Fig. 1:**
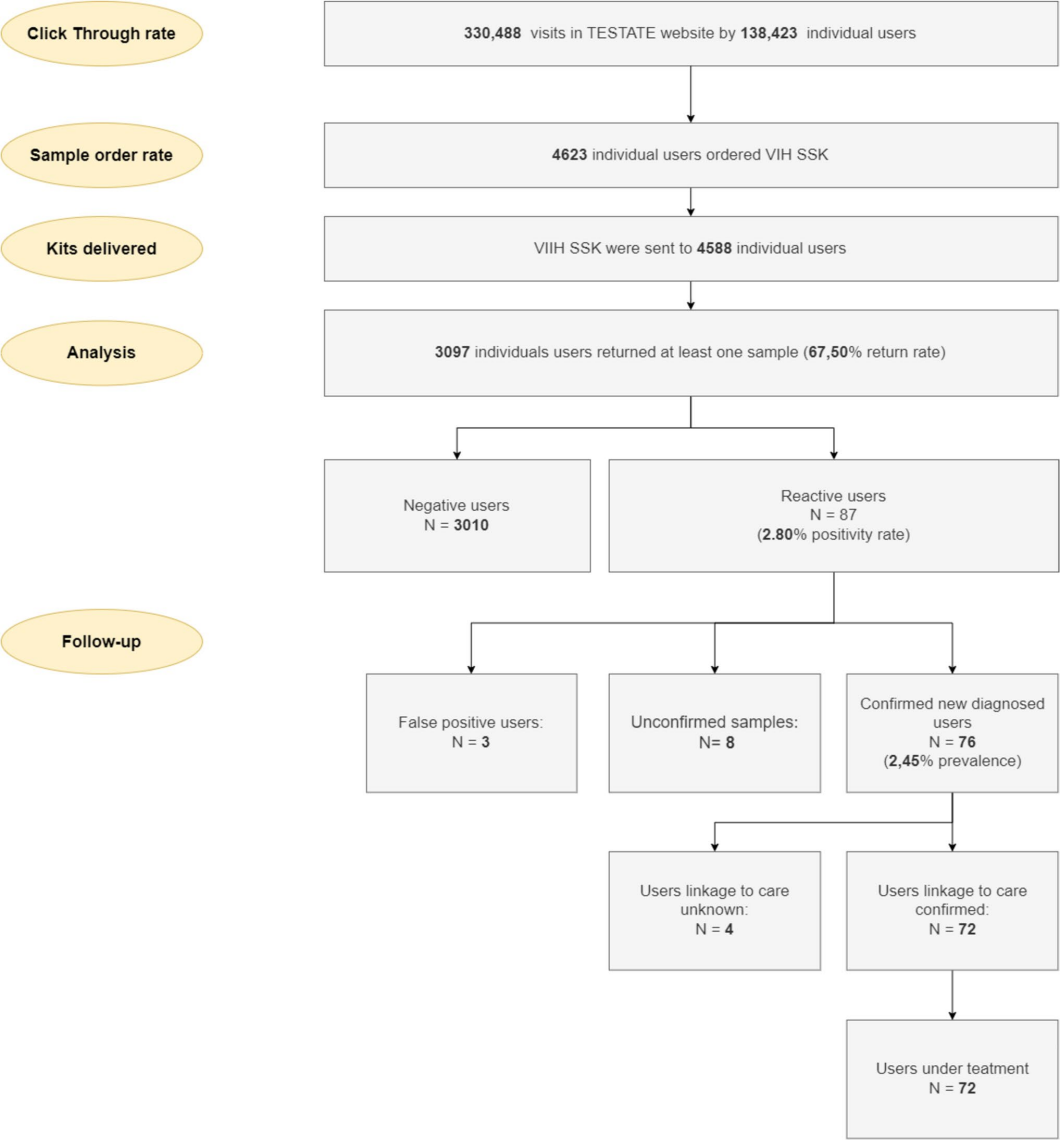
Summary diagnoses TESTATE HIV

The TESTATE website (https://testate.org/) had 330,488 visits from November 2018 to December 2021. Of these, 138,423 visits were from individual users. The response rates to the intervention are shown in Fig. 1.

### Study Population Characteristics

From November 2018 to December 2021, 4623 people requested a self-sampling kit. A kit was sent to 4588 at their home address. The characteristics of the study population are shown in Table 1. The majority were male (99.4%), the median age was 32 years [IQR 26.00;41.00] and 20.83% of the total participants were born outside Spain. About half (55.8%) had an undergraduate or postgraduate level of education. 80.82% of the participants identified themselves as gay or lesbian and 26.9% had had more than 10 or 20 sexual partners in the last year. 8.98% reside in a town with a population ≤ 10,000.

**Table 1:** Characteristics of the participants of the TESTATE intervention (overall, by obtained results and whether they are first time testers or not)

	Total N = 3097	Negative N = 3010	Reactive N = 87	p-value
	N (%)	N (%)	N (%)	
Sex				1.000
Men	3079 (99.42%)	2992 (99.40%)	87 (100.00%)	
Trans women	18 (0.58%)	18 (0.60%)	0 (0.00%)	
Age				
Median (IQR)	32.00 [26.00;41.00]	32.00 [26.00;41.00]	35.00 [29.00;41.00]	0.041
Country of birth				0.015
Spain	2453 (79.21%)	2396 (79.60%)	57 (65.52%)	
North Africa	9 (0.29%)	9 (0.30%)	0 (0.00%)	
Central and South America	380 (12.27%)	361 (11.99%)	19 (21.84%)	
North America	29 (0.94%)	27 (0.90%)	2 (2.30%)	
Asia	17 (0.55%)	16 (0.53%)	1 (1.15%)	
Eastern Europe and Russia	35 (1.13%)	32 (1.06%)	3 (3.45%)	
Western Europe	119 (3.84%)	116 (3.85%)	3 (3.45%)	
Don’t know	55 (1.78%)	53 (1.76%)	2 (2.30%)	
Level of education				0.040
Primary school	92 (2.97%)	89 (2.96%)	3 (3.45%)	
Secondary school	825 (26.64%)	791 (26.28%)	34 (39.08%)	
Vocational education	414 (13.37%)	409 (13.59%)	5 (5.75%)	
Bachelor’s or equivalent	1113 (35.94%)	1085 (36.05%)	28 (32.18%)	
Master or doctoral	614 (19.83%)	599 (19.90%)	15 (17.24%)	
Don’t know	39 (1.26%)	37 (1.23%)	2 (2.30%)	
Population city of residence				0.456
> 1 Million	947 (30.58%)	922 (30.63%)	25 (28.74%)	
500,000–1 Million	345 (11.14%)	339 (11.26%)	6 (6.90%)	
100,000–500,000	724 (23.38%)	706 (23.46%)	18 (20.69%)	
49,000–100,000	305 (9.85%)	295 (9.80%)	10 (11.49%)	
10,000–49,000	498 (16.08%)	482 (16.01%)	16 (18.39%)	
< 10,000	278 (8.98%)	266 (8.84%)	12 (13.79%)	
Previous HIV test				0.010
Yes	2545 (82.18%)	2474 (82.19%)	71 (81.61%)	
No	552 (17.82%)	536 (17.81%)	16 (18.39%)	
Time since last HIV test				0.003
< 3 months	174 (6.84%)	170 (6.87%)	4 (5.63%)	
3–6 months	942 (37.01%)	929 (37.55%)	13 (18.31%)	
6–12 months	741 (29.12%)	719 (29.06%)	22 (30.99%)	
1–5 years	612 (24.05%)	583 (23.57%)	29 (40.85%)	
> 5 years	59 (2.32%)	56 (2.26%)	3 (4.23%)	
Don’t know	17 (0.67%)	17 (0.69%)	0 (0.00%)	
Reasons for no previous HIV test				
I don’t consider myself at risk	196 (35.91%)	193 (36.01%)	3 (18.75%)	0.248
Fear of a positive result	194 (35.14%)	187 (34.89%)	7 (43.75%)	0.641
I didn’t know where to go for a test	283 (51.72%)	279 (52.05%)	4 (25.00%)	0.060
I didn’t want to go to my general practitioner	279 (50.54%)	274 (51.12%)	5 (31.25%)	0.189
I don’t have access to the health care system	11 (1.99%)	11 (2.05%)	0 (0.00%)	1.000
Other	8 (1.52%)	7 (1.37%)	1 (6.25%)	0.211
Don’t know	35 (6.34%)	32 (5.97%)	3 (18.75%)	0.531
Reason for testing				
Having had anal sex without a condom	1739 (56.15%)	1689 (56.11%)	50 (57.47%)	0.887
Having had vaginal sex without a condom	78 (2.52%)	76 (2.52%)	2 (2.30%)	1.000
Having had oral sex without a condom	1934 (62.45%)	1888 (62.72%)	46 (52.87%)	0.079
Condom breakage or slippage	279 (9.01%)	264 (8.77%)	15 (17.24%)	0.011
Regular check	1841 (59.44%)	1808 (60.07%)	33 (37.93%)	< 0.001
Knowing my state of health	1587 (51.24%)	1544 (51.30%)	43 (49.43%)	0.814
Partner HIV+	94 (3.04%)	92 (3.06%)	2 (2.30%)	1.000
Sharing injection material	6 (0.19%)	4 (0.13%)	2 (2.30%)	0.011
My partner asked me to have a test	92 (2.97%)	90 (2.99%)	2 (2.30%)	1.000
I want to stop using condoms with my partner	91 (2.94%)	89 (2.96%)	2 (2.30%)	1.000
I was in window period in my last test	114 (3.68%)	112 (3.72%)	2 (2.30%)	0.771
I have symptoms of HIV infection	19 (0.61%)	18 (0.60%)	1 (1.15%)	0.419
Other	31 (1.00%)	27 (0.90%)	4 (4.60%)	0.010
Sexual orientation				0.017
Gay	2503 (80.82%)	2429 (80.70%)	74 (85.06%)	
Heterosexual	45 (1.45%)	41 (1.36%)	4 (4.60%)	
Bisexual	528 (17.05%)	520 (17.28%)	8 (9.20%)	
Other	21 (0.68%)	20 (0.66%)	1 (1.15%)	
Number of trans men/women with whom you have had anal intercourse in the last 12 months				
None	101 (4.63%)	97 (4.58%)	4 (6.45%)	
With 1	282 (12.93%)	281 (13.26%)	1 (1.61%)	
2–4	704 (32.28%)	688 (32.47%)	16 (25.81%)	
5–9	424 (19.44%)	410 (19.35%)	14 (22.58%)	
10–20	351 (16.09%)	333 (15.71%)	18 (29.03%)	
> 20	236 (10.82%)	228 (10.76%)	8 (12.90%)	
Don’t know	83 (3.81%)	82 (3.87%)	1 (1.61%)	
Condom use last anal intercourse				1.000
Yes	1392 (46.68%)	1354 (46.67%)	38 (46.91%)	
No	1590 (53.32%)	1547 (53.33%)	43 (53.09%)	
Serostatus partner last anal intercourse				0.004
HIV negative	841 (27.17%)	827 (27.48%)	14 (16.28%)	
HIV positive with undetectable VL	128 (4.14%)	120 (3.99%)	8 (9.30%)	
HIV positive with detectable VL	6 (0.19%)	5 (0.17%)	1 (1.16%)	
HIV positive with unknown VL	11 (0.36%)	10 (0.33%)	1 (1.16%)	
Unknown serostatus	1774 (57.32%)	1725 (57.33%)	49 (56.98%)	
Don’t know	335 (10.82%)	322 (10.70%)	13 (15.12%)	
STI diagnosed in the last 5 years				
None	1905 (61.51%)	1857 (61.69%)	48 (55.17%)	0.262
Syphilis	371 (11.98%)	349 (11.59%)	22 (25.29%)	< 0.001
Gonorrhoea	448 (14.47%)	433 (14.39%)	15 (17.24%)	0.554
Chlamydia or lymphogranuloma venereum	217 (7.01%)	207 (6.88%)	10 (11.49%)	0.147
Genital warts	212 (6.85%)	208 (6.91%)	4 (4.60%)	0.531
Genital herpes	73 (2.36%)	71 (2.36%)	2 (2.30%)	1.000
Other	97 (3.16%)	91 (3.05%)	6 (6.90%)	0.055
Last STI diagnosis				0.515
Never	13 (1.14%)	13 (1.18%)	0 (0.00%)	
Last month	42 (3.70%)	41 (3.72%)	1 (2.86%)	
Last 6 months	216 (19.01%)	207 (18.80%)	9 (25.71%)	
Last 12 months	498 (43.84%)	482 (43.78%)	16 (45.71%)	
Last 5 years	183 (16.11%)	176 (15.99%)	7 (20.00%)	
> Than 5 years	92 (8.10%)	92 (8.36%)	0 (0.00%)	
Don’t know	134 (11.80%)	131 (11.89%)	3 (8.57%)	
Last STI diagnosis				0.515
Never	13 (1.14%)	13 (1.18%)	0 (0.00%)	
Last month	42 (3.70%)	41 (3.72%)	1 (2.86%)	
Last 6 months	183 (16.11%)	176 (15.99%)	7 (20.00%)	
Last 12 months	216 (19.01%)	207 (18.80%)	9 (25.71%)	
Last 5 years	498 (43.84%)	482 (43.78%)	16 (45.71%)	
> Than 5 years	92 (8.10%)	92 (8.36%)	0 (0.00%)	
On Prep				0.897
Yes	212 (6.85%)	206 (6.84%)	6 (6.90%)	
No	2823 (91.15%)	2744 (91.16%)	79 (90.80%)	
Don’t know	62 (2.00%)	60 (1.99%)	2 (2.30%)	
Repeat test through TESTATE				0.001
No	1726 (55.73%)	1662 (55.22%)	64 (73.56%)	
Yes	1371 (44.27%)	1348 (44.78%)	23 (26.44%)	
Number of repetitions				0.002
Median (IQR)	1.00 [1.00;2.00]	1.00 [1.00;2.00]	1.00 [1.00;2.00]	

November 2018–December 2021, Spain. N: 3097

In relation to STIs in the last 5 years, the most common were: gonorrhoea (14.5%) and syphilis (12.0%). 91.1% of participants were not taking PrEP. 57.3% did not know the HIV status of their last sexual partner and 17.8% reported no previous HIV test. The distribution of self-sampling kits by region in Spain is shown in Supplementary Figs. 2 and 3.

### HIV Infections and Cascade of Services

**Fig. 2:**
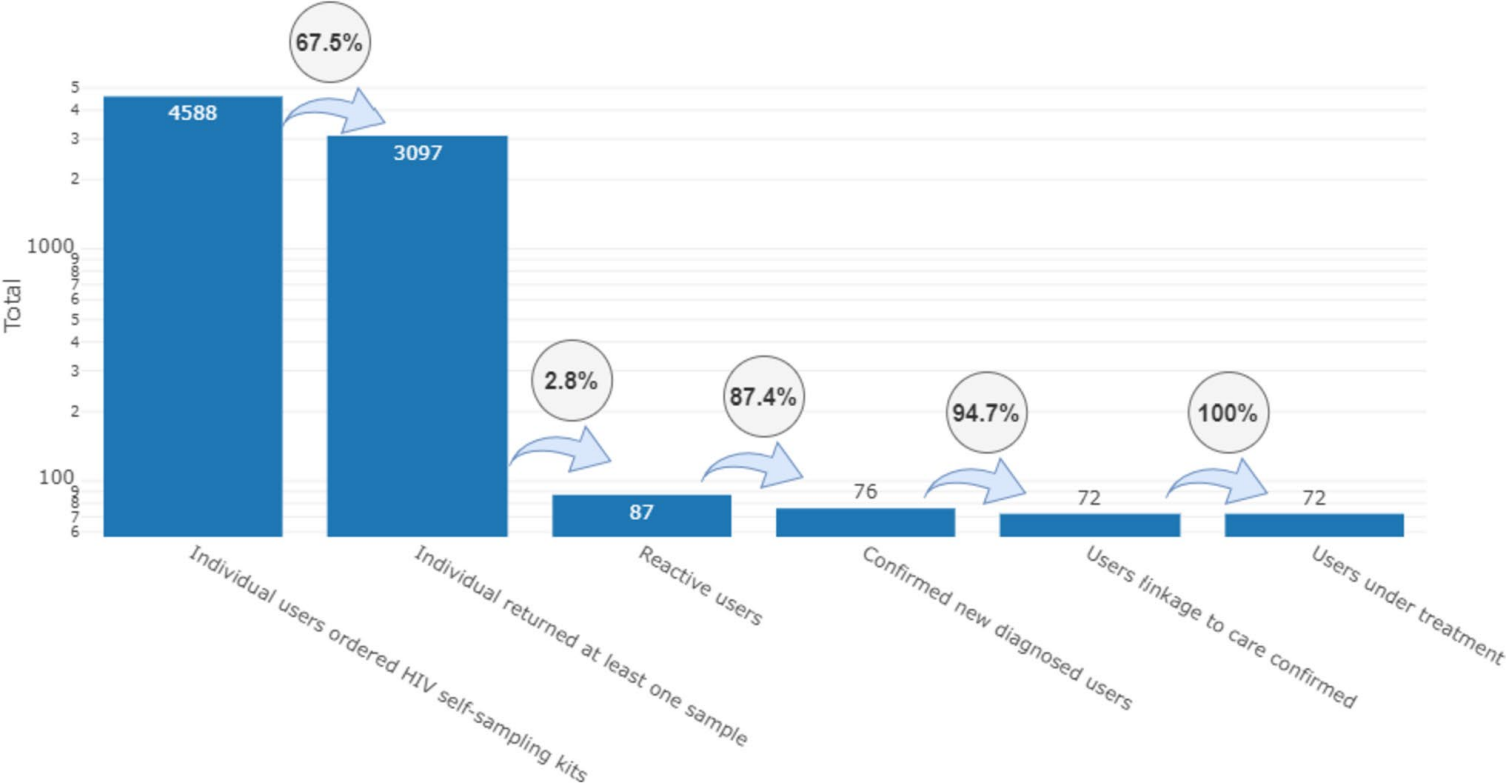
TESTATE HIV service cascade

The TESTATE HIV service cascade was estimated (Fig. 2). Of the 4588 participants who were sent a self-sampling kit, 3097 returned an oral fluid sample to the reference laboratory (67.5% return rate). Eighty-seven reactive results were detected (2.8% reactivity, 87/3097). Among those with a reactive result, 76 (87.4%, 76/87) confirmed seropositive. Among the participants who confirmed HIV-positive, 72 (94.7%, 72/76) were referred to specialised care to start treatment. All of them are currently on treatment (100%, 72/72).

We estimated an HIV prevalence of 2.45% (95% CI 1.9–3.0%). We estimate that 36 HIV tests should be performed to diagnose one case of HIV through the TESTATE HIV intervention.

Factors associated with a reactive HIV result were (Table 2): Being older than 45 years (OR 2.24; 95% CI 1.02–4.92; p = 0.045), being born in Central or South America (OR 2.35; 95% CI 1.35–4.09; p = 0.002), being born in Eastern Europe or Russia (OR 4.43; 95% CI 1.28–15.39; p = 0.019), having had a condom break or slip (OR 2.01; 95% CI 1.1–3.7; p = 0.024), having had their last anal intercourse without using a condom with an HIV-positive partner (OR 3.43, 95% CI 1.43–8.23, p = 0.006), having been diagnosed with syphilis in the last 5 years (OR 2.35, 95% CI 1.4–3.93, p = 0.001). Conversely, having regular screening tests (OR 0.41, 95% CI 0.26–0.66, p < 0.001), and being bisexual (OR 0.47, 95% CI 0.22–0.99, p = 0.050) were shown to have a protective effect*.*

**Table 2:** Associated factors to obtain a reactive result and to repeat the test through the TESTATE intervention

Reactive test		
	OR^a^ (95% CI)	p value
(Intercept)	0.01 (0.01 - 0.03)	0.000
Age		
18–25 years old	*Ref*	
26–35 years old	1.61 (0.82–3.19)	0.169
36–45 years old	1.74 (0.84–3.58)	0.134
> 45 years old	2.24 (1.02–4.92)	0.045
Country of birth		
Spain	*Ref*	
North Africa	0 (0–Inf)	0.980
Central and South America	2.35 (1.35–4.09)	0.002
North America	4.24 (0.95–18.83)	0.057
Asia	3.9 (0.49–31.11)	0.199
Eastern Europe and Russia	4.43 (1.28–15.39)	0.019
Western Europe	1.02 (0.3–3.43)	0.971
Size of city of residence		
Big city resident*	*Ref*	
Small city resident**	1.45 (0.9–2.35)	0.130
Reason for testing		
No condom breakage or displacement	*Ref*	
Yes condom breakage or displacement	2.01 (1.1–3.7)	0.024
Not having a test for a regular check	*Ref*	
Having a test for a regular check	0.41 (0.26–0.66)	0.000
Sexual orientation		
Gay	*Ref*	
Heterosexual	2.16 (0.63–7.41)	0.222
Bisexual	0.47 (0.22–0.99)	0.047
Other	2.26 (0.29–17.86)	0.438
Serostatus partner last anal sexual intercourse		
HIV negative	*Ref*	
HIV positive	3.43 (1.43–8.23)	0.005
Unknown	1.51 (0.82–2.8)	0.187
Syphilis		
No Syphilis in the last 5 years	*Ref*	
Yes Syphilis in the last 5 years	2.35 (1.4–3.93)	0.001

Repeat the test		
	IRR^b^ (95% CI)	p value
(Intercept)	2.02 (1.85–2.21)0.91 (0.82 - 1.01)	0.078
Age		
15–25 years old	*Ref*	
26–35 years old	1.09 (1.01–1.17)	0.027
36–45 years old	1.23 (1.14–1.33)	0
> 45 years old	1.36 (1.24–1.48)	0
Country of birth		
Spain	*Ref*	
North Africa	1.1 (0.69–1.74)	0.699
Central and South America	0.94 (0.87–1.02)	0.129
North America	1.33 (1.05–1.68)	0.018
Asia	1.28 (0.93–1.75)	0.127
Eastern Europe and Russia	0.89 (0.69–1.15)	0.367
Western Europe	0.84 (0.73–0.96)	0.013
Condom use in anal sex		
Yes	*Ref*	
No	1.07 (1.02–1.13)	0.009
Unknown	0.93 (0.8–1.09)	0.366
Serostatus partner last anal sexual intercourse		
HIV negative	*Ref*	
HIV positive	1.06 (0.94–1.2)	0.355
Unknown	1.05 (0.99–1.11)	0.136
Previous HIV testing		
No	*Ref*	
Yes	2.02 (1.85–2.21)	0

*OR (95% CI)* odds ratio and 95% confidence interval, *IRR (95% CI)* incidence rate ratio and 95% confidence interval

* > 1 million population; ** < 50,000 population

^a^Adjusted by: age, country of birth, city, regular check, sexual orientation, serostatus last partner, diagnosis of STI and on prep

^b^Adjusted by: age, country of birth, city, regular check, sexual orientation, serostatus last partner, diagnosis of STI and on prep

### Repeater Boxes

Of the 3097 people who returned samples; 1680 participants (54.2%) had a single test during the study period, 584 (18.9%) had two, 292 (9.4%) had three, 207 (6.7%) had four, 121 (3.9%) had five and 213 had six or more (6.9%). Repeat testing was done with a minimum interval of three months between tests.

The median number of tests performed through the TESTATE intervention was 1 (IQR: 1–2) (Table 1). The negative binomial model showed that the likelihood of repeat testing through the TESTATE intervention increases for the following variables: being aged 25 years and older; being born in North America (OR 1.33; 95% CI 1.05–1.68; p = 0.018); having anal intercourse without a condom (OR 1.07; 95% CI 1.02–1.13; p = 0.010) or having had a previous HIV test (OR 2.02; 95% CI 1.85–2.21; p < 0.001) (Table 2). Conversely, being born in Western Europe (OR 0.84; 95% CI 0.73–0.96; p = 0.013) was shown to have a protective effect.

64 of the reactive individuals were reactive on the first test and 23 incident cases were detected among repeat testers, of which 20 were confirmed. The estimated incidence was 1.00 confirmed cases per 100 person-years.

### Satisfaction Section

A total of 404 responses to the anonymous satisfaction survey were collected (Table 3). In terms of participant satisfaction, on a scale of one to five, the average score for evaluating the experience was 4.78. The most identified advantages were convenience (94.06%), privacy (91.58%) and being free of charge (84.9%); and the most identified disadvantages were not having the results immediately (38.61%) and not having emotional support when receiving the result (37.13%). The preferred way of receiving the result was via the web (75.99%) and followed by SMS (12.13%). 96.29% said they would repeat the experience and almost all participants (98.01%) would recommend testing with TESTATE HIV.

**Table 3:** Satisfaction, willingness, perceived advantages and disadvantages of the TESTATE intervention

	N = 404	%
Would repeat the experience		
Yes	389	96.29
No	3	0.74
I’m not sure	9	2.23
Don’t know	3	0.74
Would recommend it to a friend		
Yes	396	98.02
No	1	0.24
I’m not sure	6	1.48
Don’t know	1	0.24
Identified advantages		
Convenience	380	94.06
Privacy and confidentiality	370	91.58
Free test	343	84.9
Explanations are not required	304	75.25
Contributes to the normalization of the test	270	66.83
No prescription required	251	62.13
Blood not needed	234	57.92
Enables me to take control of healthcare	142	35.15
Identified disadvantages		
Not having the results immediately	156	38.61
Risk of sample loss in shipping	147	36.39
Not having emotional support	150	37.13
Need of confirmation	121	29.95
Long time to know the result	133	32.92
Preferred way to receive the test result		
Website	307	75.99
SMS	49	12.13
Telephone	8	1.98
Face to face with a physician	15	3.71
At an NGO	6	1.49
Don’t know	19	4.70
	Score	
Assessment of the experience (1–5)	4.78	

November 2018–December 2021, Spain. N: 404

## Discussion

The TESTATE HIV study demonstrates that the provision of self-sampling kits (SSKs) for HIV testing in oral fluid and online consultation of results in Spain is feasible, as it is in other European states [37, 38]. The intervention has enabled access to HIV testing for people at risk of acquiring HIV infection: 91.1% of participants were not on PrEP, 57.3% did not know the HIV status of their last sexual partner and 26.9% had had 10 or more than 20 sexual partners in the last year. In relation to STIs in the last 5 years, the most common were: gonorrhoea (14.5%) and syphilis (11.9%). In addition, 552 participants who had never been tested for HIV had access to testing for the first time (17.8%) and 283 participants (9.14% of all participants) who had never been tested had not been tested because they did not know where to go for testing. The EMIS study [10] in 2017 already reported that lack of knowledge of where to go for HIV testing was most prevalent among those living in cities of less than 100,000 inhabitants, those under 25 years of age and those with lower educational attainment.

Postal SSKs have been shown to increase not only STI and HIV screening, but also the rate of positive tests compared to tests collected by general practitioners [39]. In TESTATE HIV, a high reactivity (2.8%) and HIV infection prevalence of 2.45% (95%CI 1.9–3.0) was obtained. From January to October 2021, the Spanish Network of Community HIV Screening Programmes (REDCOVIH) estimated a reactivity of 1.86% [40] and the COBATEST Network (a platform for monitoring and evaluating community-based HIV testing and counselling practices in Europe) estimated a prevalence in GBMSM of 1.28% [41], both lower than those found in the TESTATE HIV study. Previous SSK postal studies at the European level have had lower reactivity in their GBMSM population [37, 38].

In 2018 the UK national HIV self-sampling service [37] concluded that a low-cost testing service can complement current service provision to key populations, such as GBMSM and TW. It is considered that a screening prevalence above 0.2% would be cost-effective [42]. In addition, only 36 tests would need to be performed through the intervention to find a confirmed HIV diagnosis.

The TESTATE HIV study had high rates of confirmation (87.4%) and linkage to care to initiate treatment (94.7%), similar to previous studies [24, 25], although slightly lower than the percentage of individuals with a reactive screening test who were linked to care in a network of CBVCT services in Spain (87.4% vs. 89.0%).

Increasing the use and frequency of HIV testing is a public health priority as an integral part of combination HIV prevention, reflected in the ambitious UNAIDS 95-95-95 target of 95% of people living with HIV knowing their HIV status, 95% of these receiving antiretroviral treatment and 95% of those receiving antiretroviral treatment being virally suppressed by 2030 [43]. The TESTATE HIV study achieves the UNAIDS 95-95-95 targets, as can be seen in the TESTATE HIV service cascade where 94.7% of HIV-positive people detected in the study have been successfully linked to the health system and 100% are receiving treatment.

TESTATE HIV is the first SSK intervention for HIV on record in Spain. Unlike other studies, TESTATE HIV employs oral fluid sampling, which offers convenient and painless collection and very little risk of contamination during collection and transport, making it ideal for self-sampling [16, 24]. It had high levels of satisfaction and willingness on the part of the target population and high efficiency (in terms of number of tests requested, samples returned, reactivity, confirmation and linkage rates to care). These results are consistent with similar previous studies [23–25]. 96.29% said they would repeat the experience and almost all participants (98.01%) would recommend testing with TESTATE HIV. The overall rating of the experience by participants was 4.78 out of 5. In addition, the TESTATE strategy has proven to be an appropriate periodic screening tool for those considered at risk of infection, almost half of the participants participated more than once in the project and 20 incident cases have been confirmed (estimated incidence of 1.00 per 100 person-years).

Complementary testing modalities to existing testing strategies, such as self-sampling (HIV, hepatitis, papilloma and STIs), are important options for diversifying and optimising access to testing that should be regulated and made available as part of national policy and practice. E-health testing, such as TESTATE, can circumvent the inconvenience and stigma associated with face-to-face services and could expand access to populations that do not use these services [26]. In addition, the SSK approach can help eliminate geographical inequalities associated with access to screening, as demonstrated by the MemoDespistages programme in France [44]. In TESTATE, 8.98% of respondents reside in a town with a population < 10,000.

This intervention targeted GBMSM and TW, two of the populations most affected by the HIV epidemic in Spain. The intervention was disseminated online through the main location-based real-time dating apps operating in Spain, which are very popular in the GBMSM community. Dating apps could play an important role as a tool for implementing HIV prevention and screening interventions targeting GBMSM, as they offer the possibility of disseminating the intervention to a wider population and the possibility of using technologies with which the population is familiar [45]. The use of apps serves as a bridge to reach hard-to-reach populations that do not use conventional health resources, as seen in other studies in Barcelona [27, 32].

Our study has several limitations. The study worked with an opportunistic sample, so the study population is not representative of all GBMSM and TW dating app users in Spain. The specificity of an HIV test on oral fluid is lower than on a blood sample. This may lead to some false reactive results. In our study they were detected (3/3097, 0.09%), however, this proportion is lower than that observed in previous studies [24]. It was not possible to distinguish whether the 32.5% of participants who failed return the sample to the laboratory did not send it back because they did not want to, or because it was lost during shipment. We only have 404 responses from the satisfaction survey and could not distinguish if the same participant answered the survey more than once, as it was an anonymous survey.

The study also has strengths. Our intervention has proven to be feasible and could be consolidated as a service and easily adapted to include other infections such as chlamydia, gonorrhoea, papilloma [46] and hepatitis C [47]; as well as being used for regular follow-up of PrEP users. The website is easily adaptable for self-sampling or self-testing for new outbreaks of infections, such as Mpox [48] or Shigella [49]. And it could also be adapted for automatic three-monthly periodical submission of SSKs for HIV and other STIs, as was done in France [44].

In conclusion, TESTATE HIV has demonstrated that the delivery of oral fluid SSKs for HIV screening in GBMSM and TW is feasible and viable in the Spanish state. Although an economic evaluation is needed, the scientific literature affirms that it would be cost-effective. In addition, it would bring the test closer to rural areas where there are fewer medical resources available. In a pandemic situation, such as that experienced by COVID-19 or in other situations of population closure, it makes more sense than ever to increase efforts to improve access and facilitate testing as it can reduce the healthcare burden on primary care services and STI consultations, while also reducing people’s mobility. Finally, it could be an adaptable tool for different STIs and additionally could be used for regular follow-up of participants and also for users of PrEP.

## Supplementary Information

Below is the link to the electronic supplementary material.

Supplementary file1 (DOCX 199 kb)
