# Correction: An Online HIV Self‑Sampling Strategy for Gay, Bisexual and Other Men Who Have Sex with Men and Trans Women in Spain

**DOI:** 10.1007/s10900-024-01387-w

**Published:** 2024-10-15

**Authors:** Héctor Martinez‑Riveros, Yesika Díaz, Marcos Montoro‑Fernandez, Sergio Moreno‑Fornes, Victoria González, Esteve Muntada, Pol Romano‑deGea, Rafael Muñoz, Juan Hoyos, Jordi Casabona, Cristina Agustí

**Affiliations:** 1 Doctorate Program in Methodology of Biomedical Research and Public Health, Department of Paediatrics, Obstetrics and Gynaecology and Preventive Medicine, Universitat Autònoma de Barcelona, Badalona, Spain; 2 Centre for Epidemiological Studies on Sexually Transmitted Infections and AIDS of Catalonia (CEEISCAT), Ministry of Health of the Government of Catalonia, Badalona, Spain; 3 Germans Trias i Pujol Research Institute (IGTP), Campus Can Ruti, Badalona, Spain; 4 Fundació Institut d’Investigació en Ciències de la Salut Germans Trias i Pujol (IGTP), Edifici Muntanya, Carretera de Can Ruti, Camí de les Escoles s/n, Badalona, 08916 Spain; 5 Biomedical Research Center Network for Epidemiology and Public Health (CIBERESP), Instituto de Salud Carlos III, Madrid, Spain; 6 Microbiology Service, Clinical Laboratory Metropolitana Nord, Badalona, Barcelona Spain; 7 Early Detection of Cancer Research Group, EPIBELL Program, Bellvitge Biomedical Research Institute, L’Hospitalet de Llobregat, Barcelona, Spain; 8 Independent consultant, Madrid, Spain; 9 Department of Genetics and Microbiology, Autonomous University of Barcelona, Badalona, Spain

**Correction to: Journal of Community Health**

10.1007/s10900-023-01311-8

In Table 1 of this article, the data in the column and row were mistakenly published with errors. The correct table is given below.

**Table 1:** Characteristics of the participants of the TESTATE intervention (overall, by obtained results and whether they are first time testers or not). November 2018 - December 2021, Spain. N: 3,097

	Total *N* = 3097		Negative *N* = 3010	Reactive *N* = 87	*p*-value
	*N* (%)		*N* (%)	*N* (%)	
**Sex**					1.000
Men	3079 (99.42%)		2992 (99.40%)	87 (100.00%)	
Trans Women	18 (0.58%)		18 (0.60%)	0 (0.00%)	
**Age**					
Median (IQR)	32.00 [26.00;41.00]		32.00 [26.00;41.00]	35.00 [29.00;41.00]	0.041
**Country of birth**					0.015
Spain	2453 (79.21%)		2396 (79.60%)	57 (65.52%)	
North Africa	9 (0.29%)		9 (0.30%)	0 (0.00%)	
Central and South America	380 (12.27%)		361 (11.99%)	19 (21.84%)	
North America	29 (0.94%)		27 (0.90%)	2 (2.30%)	
Asia	17 (0.55%)		16 (0.53%)	1 (1.15%)	
Eastern Europe and Russia	35 (1.13%)		32 (1.06%)	3 (3.45%)	
Western Europe	119 (3.84%)		116 (3.85%)	3 (3.45%)	
Don’t know	55 (1.78%)		53 (1.76%)	2 (2.30%)	
**Level of education**					0.040
Primary school	92 (2.97%)		89 (2.96%)	3 (3.45%)	
Secondary school	825 (26.64%)		791 (26.28%)	34 (39.08%)	
Vocational education	414 (13.37%)		409 (13.59%)	5 (5.75%)	
Bachelor’s or equivalent	1113 (35.94%)		1085 (36.05%)	28 (32.18%)	
Master or doctoral	614 (19.83%)		599 (19.90%)	15 (17.24%)	
Don’t know	39 (1.26%)		37 (1.23%)	2 (2.30%)	
**Population city of residence**					0.456
> 1 Million	947 (30.58%)		922 (30.63%)	25 (28.74%)	
500,000–1 Million	345 (11.14%)		339 (11.26%)	6 (6.90%)	
100,000–500,000	724 (23.38%)		706 (23.46%)	18 (20.69%)	
49,000–100,000	305 (9.85%)		295 (9.80%)	10 (11.49%)	
10,000–49,000	498 (16.08%)		482 (16.01%)	16 (18.39%)	
< 10,000	278 (8.98%)		266 (8.84%)	12 (13.79%)	
**Previous HIV test**					0.010
Yes	2545 (82.18%)		2474 (82.19%)	71 (81.61%)	
No	552 (17.82%)		536 (17.81%)	16 (18.39%)	
**Time since last HIV test**					0.003
<3 months	174 (6.84%)		170 (6.87%)	4 (5.63%)	
3–6 months	942 (37.01%)		929 (37.55%)	13 (18.31%)	
6–12 months	741 (29.12%)		719 (29.06%)	22 (30.99%)	
1–5 years	612 (24.05%)		583 (23.57%)	29 (40.85%)	
> 5 years	59 (2.32%)		56 (2.26%)	3 (4.23%)	
Don’t know	17 (0.67%)		17 (0.69%)	0 (0.00%)	
**Reasons for no previous HIV test**					
I don’t consider myself at risk	196 (35.91%)		193 (36.01%)	3 (18.75%)	0.248
Fear of a positive result	194 (35.14%)		187 (34.89%)	7 (43.75%)	0.641
I didn’t know where to go for a test	283 (51.72%)		279 (52.05%)	4 (25.00%)	0.060
I didn’t want to go to my general practitioner	279 (50.54%)		274 (51.12%)	5 (31.25%)	0.189
I don’t have access to the health care system	11 (1.99%)		11 (2.05%)	0 (0.00%)	1.000
Other	8 (1.52%)		7 (1.37%)	1 (6.25%)	0.211
Don’t know	35 (6.34%)		32 (5.97%)	3 (18.75%)	0.531
**Reason for testing**					
Having had anal sex without a condom	1739 (56.15%)		1689 (56.11%)	50 (57.47%)	0.887
Having had vaginal sex without a condom	78 (2.52%)		76 (2.52%)	2 (2.30%)	1.000
Having had oral sex without a condom	1934 (62.45%)		1888 (62.72%)	46 (52.87%)	0.079
Condom breakage or slippage	279 (9.01%)		264 (8.77%)	15 (17.24%)	0.011
Regular check	1841 (59.44%)		1808 (60.07%)	33 (37.93%)	< 0.001
Knowing my state of health	1587 (51.24%)		1544 (51.30%)	43 (49.43%)	0.814
Partner HIV+	94 (3.04%)		92 (3.06%)	2 (2.30%)	1.000
Sharing injection material	6 (0.19%)		4 (0.13%)	2 (2.30%)	0.011
My partner asked me to have a test	92 (2.97%)		90 (2.99%)	2 (2.30%)	1.000
I want to stop using condoms with my partner	91 (2.94%)		89 (2.96%)	2 (2.30%)	1.000
I was in window period in my last test	114 (3.68%)		112 (3.72%)	2 (2.30%)	0.771
I have symptoms of HIV infection	19 (0.61%)		18 (0.60%)	1 (1.15%)	0.419
Other	31 (1.00%)		27 (0.90%)	4 (4.60%)	0.010
**Sexual orientation**					0.017
Gay	2503 (80.82%)		2429 (80.70%)	74 (85.06%)	
Heterosexual	45 (1.45%)		41 (1.36%)	4 (4.60%)	
Bisexual	528 (17.05%)		520 (17.28%)	8 (9.20%)	
Other	21 (0.68%)		20 (0.66%)	1 (1.15%)	
**Number of trans men/women with whom you have had anal intercourse in the last 12 months**					
None	101 (4.63%)		97 (4.58%)	4 (6.45%)	
With 1	282 (12.93%)		281 (13.26%)	1 (1.61%)	
2–4	704 (32.28%)		688 (32.47%)	16 (25.81%)	
5–9	424 (19.44%)		410 (19.35%)	14 (22.58%)	
10–20	351 (16.09%)		333 (15.71%)	18 (29.03%)	
>20	236 (10.82%)		228 (10.76%)	8 (12.90%)	
Don’t know	83 (3.81%)		82 (3.87%)	1 (1.61%)	
**Condom use last anal intercourse**					1.000
Yes	1392 (46.68%)		1354 (46.67%)	38 (46.91%)	
No	1590 (53.32%)		1547 (53.33%)	43 (53.09%)	
**Serostatus partner last anal intercourse**					0.004
HIV negative	841 (27.17%)		827 (27.48%)	14 (16.28%)	
HIV positive with undetectable VL	128 (4.14%)		120 (3.99%)	8 (9.30%)	
HIV positive with detectable VL	6 (0.19%)		5 (0.17%)	1 (1.16%)	
HIV positive with unknown VL	11 (0.36%)		10 (0.33%)	1 (1.16%)	
Unknown serostatus	1774 (57.32%)		1725 (57.33%)	49 (56.98%)	
Don’t know	335 (10.82%)		322 (10.70%)	13 (15.12%)	
**STI diagnosed in the last 5 years**					
None	1905 (61.51%)		1857 (61.69%)	48 (55.17%)	0.262
Syphilis	371 (11.98%)		349 (11.59%)	22 (25.29%)	< 0.001
Gonorrhoea	448 (14.47%)		433 (14.39%)	15 (17.24%)	0.554
Chlamydia or lymphogranuloma venereum	217 (7.01%)		207 (6.88%)	10 (11.49%)	0.147
Genital warts	212 (6.85%)		208 (6.91%)	4 (4.60%)	0.531
Genital herpes	73 (2.36%)		71 (2.36%)	2 (2.30%)	1.000
Other	97 (3.16%)		91 (3.05%)	6 (6.90%)	0.055
**Last STI diagnosis**					0.515
Never	13 (1.14%)		13 (1.18%)	0 (0.00%)	
Last month	42 (3.70%)		41 (3.72%)	1 (2.86%)	
Last 6 months	183 (16.11%)		176 (15.99%)	7 (20.00%)	
Last 12 months	216 (19.01%)		207 (18.80%)	9 (25.71%)	
Last 5 years	498 (43.84%)		482 (43.78%)	16 (45.71%)	
> than 5 years	92 (8.10%)		92 (8.36%)	0 (0.00%)	
Don’t know	134 (11.80%)		131 (11.89%)	3 (8.57%)	
**Last STI diagnosis**					0.515
Never	13 (1.14%)		13 (1.18%)	0 (0.00%)	
Last month	42 (3.70%)		41 (3.72%)	1 (2.86%)	
Last 6 months	183 (16.11%)		176 (15.99%)	7 (20.00%)	
Last 12 months	216 (19.01%)		207 (18.80%)	9 (25.71%)	
Last 5 years	498 (43.84%)		482 (43.78%)	16 (45.71%)	
> than 5 years	92 (8.10%)		92 (8.36%)	0 (0.00%)	
**On Prep**					0.897
Yes	212 (6.85%)		206 (6.84%)	6 (6.90%)	
No	2823 (91.15%)		2744 (91.16%)	79 (90.80%)	
Don’t know	62 (2.00%)		60 (1.99%)	2 (2.30%)	
**Repeat test through TESTATE**					0.001
No	1726 (55.73%)		1662 (55.22%)	64 (73.56%)	
Yes	1371 (44.27%)		1348 (44.78%)	23 (26.44%)	
**Number of repetitions**					0.002
Median (IQR)	1.00 [1.00;2.00]		1.00 [1.00;2.00]	1.00 [1.00;2.00]	

In addition,

In page 535, under abstract section, in line 9, the word “45.8%”should have been “44.3”%.

In page 538, in column 2, second paragraph, in line 10, “16.01%” should have been “8.98%”.

In page 542, in column 2, in line 2, the sentence “having anal intercourse without anal intercourse (OR 1.33; 95% CI 1.05–1.68; p = 0.018)0.05–1.68; p = 0.018)” has been deleted from the article.

In page 544, in column 2, line 8, the word “518” should have been “552” and in line 10, the sentence “(16.7%) and 270 participants (8.71%” should have been “(17.8%) and 283 participants 9.14%”.

In page 545, in column 2, fourth paragraph, in line 12, the word “18.39%” should have been “8.98%”.

The original article has been corrected.

