# Supplementary material for: An Online HIV Self-Sampling Strategy for Gay, Bisexual and Other Men Who Have Sex with Men and Trans Women in Spain
Source: J Community Health. 2023 Dec 23;49(3):535–48. doi: 10.1007/s10900-023-01311-8 (PMC10981614; doi:10.1007/s10900-023-01311-8)
Supplement: Supplementary file 1 — Supplementary file1 (DOCX 199 kb) [file 10900_2023_1311_MOESM1_ESM.docx]

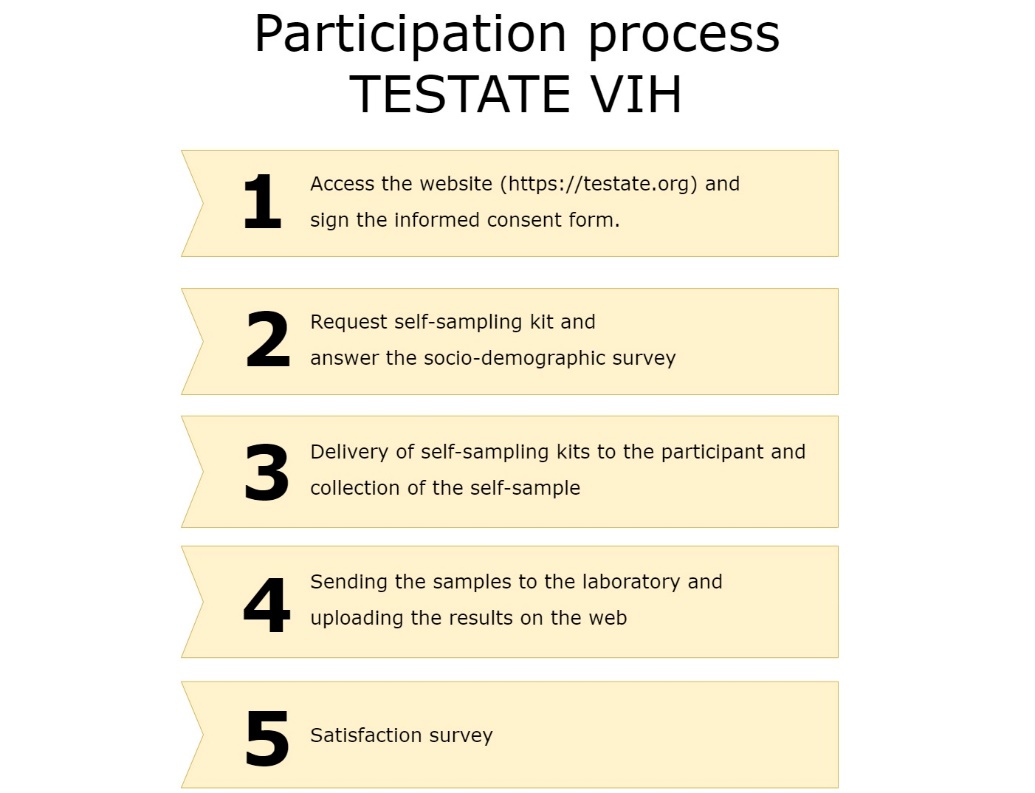


*Supplementary figure 1. TESTATE HIV participation process*


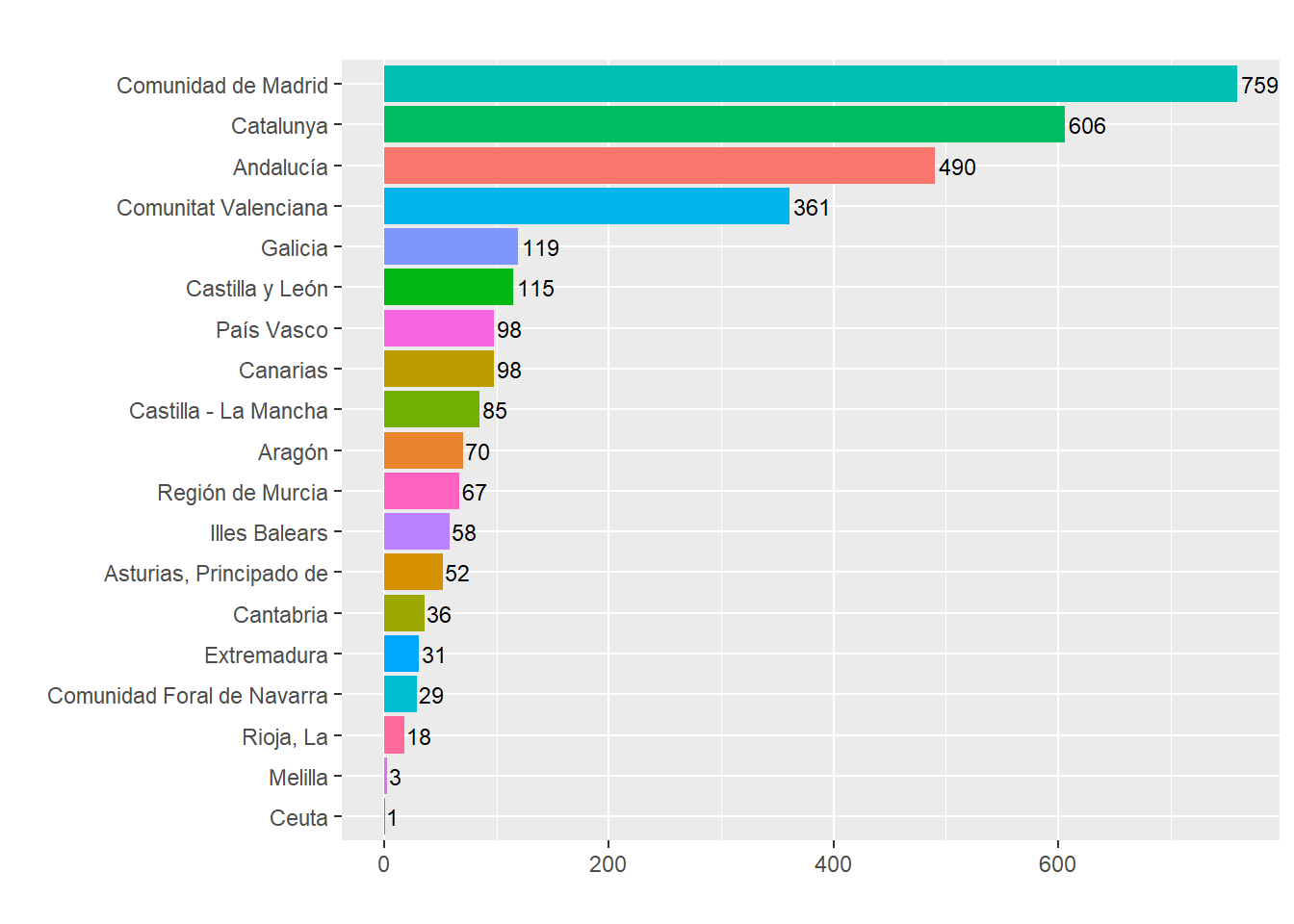
*Supplementary figure 2. Number of TESTATE HIV participants by autonomous community*


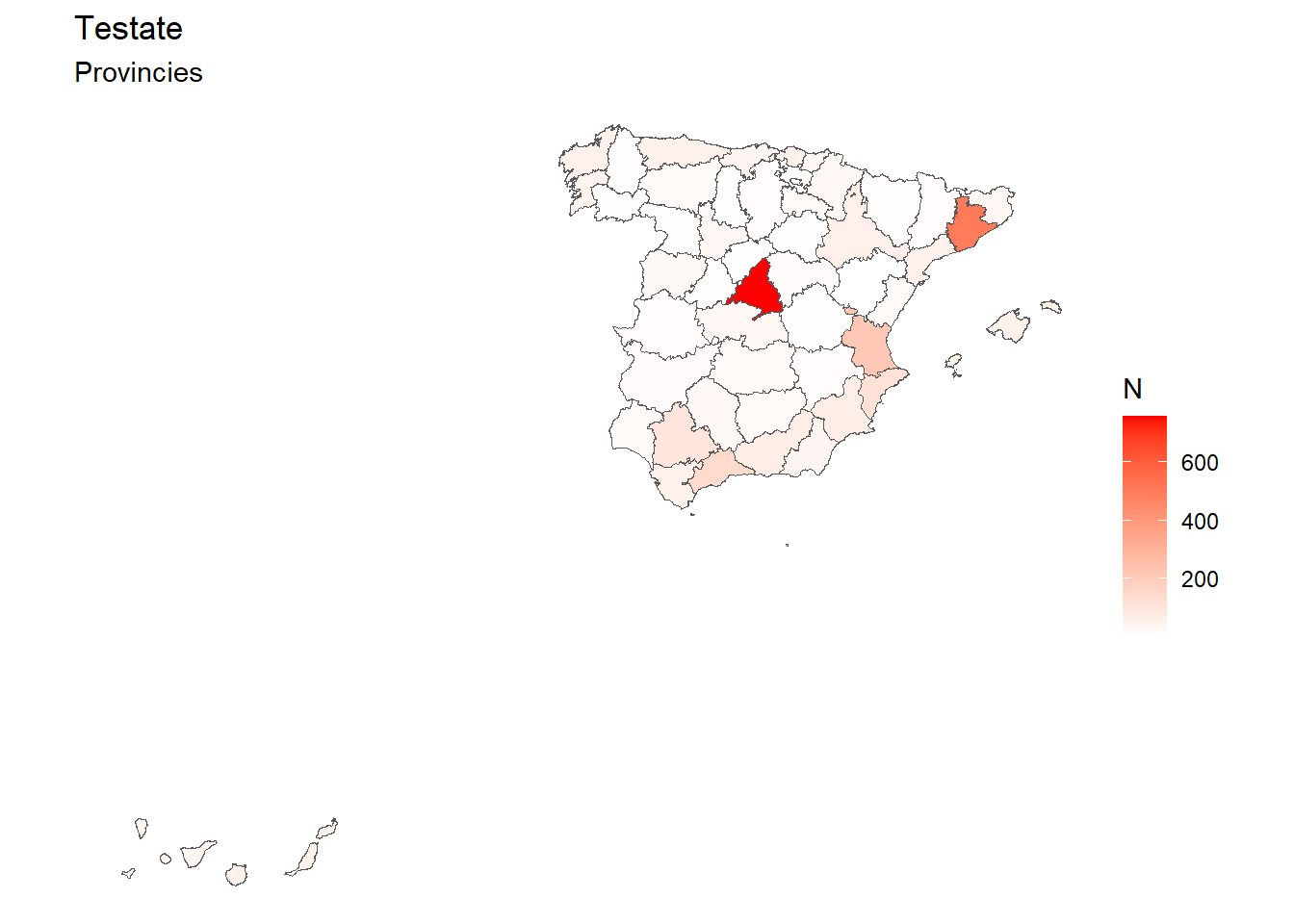


*Supplementary figure 3. Map of TESTATE HIV participation by province in Spain*
